# Supplementary material for: Parental compliance and reasons for COVID-19 Vaccination among American children
Source: PLOS Digit Health. 2023 Apr 12;2(4):e0000147. doi: 10.1371/journal.pdig.0000147 (PMC10096220; doi:10.1371/journal.pdig.0000147)
Supplement: S1 Table — (DOCX) [file pdig.0000147.s002.docx]

S1 Table. Multivariate Results for Parents with Children Under 5 and No Children Under 5

|  | **Willingness to Vaccinate Children** | |
| --- | --- | --- |
|  | **Parents with Children 5-17 and No Children Under 5** | **Parents with Children 5-17 and Children Under 5** |
|  | **Odds Ratio (95% confidence interval)** | **Odds Ratio (95% confidence interval)** |
| Gender |  |  |
| Female | — | — |
| Male | 1.16 (1.08, 1.25)*** | 1.17 (1.09, 1.24)*** |
| Transgender or Nonbinary | 0.71 (0.53, 0.97)* | 0.56 (0.43, 0.72)*** |
| Age |  |  |
| 18-29 years | — | — |
| 30-39 years | 0.98 (0.85, 1.14) | 1.02 (0.91, 1.14) |
| 40-49 years | 1.30 (1.12, 1.51)*** | 1.44 (1.27, 1.62)*** |
| 50-64 years | 1.50 (1.27, 1.76)*** | 1.61 (1.40, 1.85)*** |
| 65+ years | 1.30 (0.99, 1.70) | 1.33 (1.06, 1.67)* |
| Household Income |  |  |
| Under $49,999 | — | — |
| $50,000-$99,999 | 0.75 (0.69, 0.83)*** | 0.76 (0.70, 0.82)*** |
| Over $100,000 | 0.72 (0.65, 0.79)*** | 0.68 (0.62, 0.74)*** |
| Race/Ethnicity |  |  |
| White, not Hispanic | — | — |
| Hispanic | 1.84 (1.68, 2.02)*** | 1.81 (1.67, 1.97)*** |
| Black | 1.61 (1.43, 1.81)*** | 1.57 (1.42, 1.75)*** |
| Asian | 3.07 (2.56, 3.71)*** | 3.12 (2.65, 3.69)*** |
| Other | 1.12 (0.95, 1.33) | 1.17 (1.00, 1.35)* |
| Education |  |  |
| High School or Less | — | — |
| Some College | 0.80 (0.73, 0.87)*** | 0.80 (0.74, 0.86)*** |
| College Graduate | 0.97 (0.87, 1.07) | 0.96 (0.88, 1.06) |
| Employment Status |  |  |
| Employed | — | — |
| Unemployed | 1.58 (1.43, 1.74)*** | 1.49 (1.37, 1.62)*** |
| Health Insurance |  |  |
| Insured | — | — |
| Uninsured | 1.23 (1.09, 1.40)** | 1.20 (1.07, 1.34)** |
| Self Reported Health |  |  |
| Fair/Poor | — | — |
| Good | 1.05 (0.91, 1.20) | 1.04 (0.92, 1.18) |
| Very good | 1.05 (0.92, 1.20) | 1.01 (0.89, 1.13) |
| Excellent | 0.99 (0.86, 1.13) | 0.96 (0.85, 1.08) |
| Religious Status |  |  |
| Religious | — | — |
| Atheist/Agnostic | 1.35 (1.24, 1.47)*** | 1.36 (1.26, 1.47)*** |
| Have Child Age 5 to 11 Years |  |  |
| No | — | — |
| Yes | 0.51 (0.47, 0.56)*** | 0.50 (0.46, 0.54)*** |
| Have Child Age 12 to 15 Years |  |  |
| No | — | — |
| Yes | 1.05 (0.98, 1.14) | 1.09 (1.02, 1.17)** |
| Have Child Age 16 to 17 Years |  |  |
| No | — | — |
| Yes | 1.34 (1.23, 1.46)*** | 1.35 (1.25, 1.47)*** |
| Political Party Affiliation |  |  |
| Republican | — | — |
| Democrat | 4.22 (3.82, 4.66)*** | 4.08 (3.74, 4.45)*** |
| Independent | 1.55 (1.44, 1.68)*** | 1.62 (1.51, 1.74)*** |
| Parent Vaccination Status |  |  |
| Unvaccinated | — | — |
| Partially Vaccinated | 11.9 (10.6, 13.3)*** | 11.8 (10.7, 13.0)*** |
| Fully Vaccinated | 19.9 (18.2, 21.7)*** | 19.9 (18.4, 21.5)*** |
| Fully Vaccinated and Boosted | 106 (93.9, 120)*** | 109 (97.5, 122)*** |

*p<.05; **p<.01; ***p<.001

We remove parents with any children under 5, even if they have children ages 5-17. We do this in order to remove any ambiguity when parents list reasons for their hesitancy to vaccinate their children. While this could inadvertently act as a control for family size, restricting to parents without children under 5 does not seem to change results significantly, suggesting that we are not changing results significantly.
