# Supplementary material for: Parental compliance and reasons for COVID-19 Vaccination among American children
Source: PLOS Digit Health. 2023 Apr 12;2(4):e0000147. doi: 10.1371/journal.pdig.0000147 (PMC10096220; doi:10.1371/journal.pdig.0000147)
Supplement: S2 Table — (DOCX) [file pdig.0000147.s003.docx]

S2 Table. Reasons for Refusal by Cluster

|  | ***All Unwilling Parents** | **Cluster 1** | | | **Cluster 2** | | | **Cluster 3** | | |
| --- | --- | --- | --- | --- | --- | --- | --- | --- | --- | --- |
|  | **Overall, N = 9,998** | **Unvaccinated, N = 2,342** | **Vaccinated**, N = 847** | **p-value†** | **Unvaccinated, N = 3,400** | **Vaccinated**, N = 2,176** | **p-value†** | **Unvaccinated, N = 733** | **Vaccinated**, N = 500** | **p-value†** |
| **Reason** |  |  |  |  |  |  |  |  |  |  |
| Child received flu vaccine and is protected | 55/9,998 (0.5%) | 33/2,342 (1.4%) | 0/847 (0%) | 0.015 | 20/3,400 (0.6%) | 0/2,176 (0%) | 0.026 | 2/733 (0.3%) | 0/500 (0%) | 0.13 |
| Vaccine too new | 4,350/9,998 (44%) | 1,912/2,342 (82%) | 645/847 (76%) | 0.011 | 454/3,400 (13%) | 334/2,176 (15%) | 0.13 | 605/733 (83%) | 400/500 (80%) | 0.37 |
| Child too young | 2,295/9,998 (23%) | 808/2,342 (35%) | 373/847 (44%) | <0.001 | 357/3,400 (10%) | 386/2,176 (18%) | <0.001 | 202/733 (28%) | 169/500 (34%) | 0.077 |
| Let child decide when older | 2,040/9,998 (20%) | 790/2,342 (34%) | 292/847 (35%) | 0.76 | 377/3,400 (11%) | 303/2,176 (14%) | 0.024 | 170/733 (23%) | 109/500 (22%) | 0.66 |
| Side Effects | 4,673/9,998 (47%) | 1,946/2,342 (83%) | 686/847 (81%) | 0.31 | 502/3,400 (15%) | 450/2,176 (21%) | <0.001 | 651/733 (89%) | 438/500 (88%) | 0.68 |
| COVID-19 threat exaggerated | 2,403/9,998 (24%) | 1,434/2,342 (61%) | 476/847 (56%) | 0.053 | 266/3,400 (7.8%) | 135/2,176 (6.2%) | 0.095 | 52/733 (7.1%) | 40/500 (8.0%) | 0.65 |
| Lack trust in government | 3,963/9,998 (40%) | 2,175/2,342 (93%) | 746/847 (88%) | <0.001 | 680/3,400 (20%) | 228/2,176 (10%) | <0.001 | 101/733 (14%) | 34/500 (6.7%) | 0.004 |
| Lack trust in scientists | 3,385/9,998 (34%) | 1,924/2,342 (82%) | 690/847 (81%) | 0.72 | 446/3,400 (13%) | 201/2,176 (9.2%) | 0.001 | 82/733 (11%) | 42/500 (8.4%) | 0.24 |
| Vaccine development too political | 3,546/9,998 (35%) | 2,016/2,342 (86%) | 738/847 (87%) | 0.57 | 365/3,400 (11%) | 245/2,176 (11%) | 0.67 | 109/733 (15%) | 73/500 (15%) | 0.88 |
| Child already had COVID-19 | 2,525/9,998 (25%) | 880/2,342 (38%) | 281/847 (33%) | 0.082 | 549/3,400 (16%) | 406/2,176 (19%) | 0.079 | 264/733 (36%) | 146/500 (29%) | 0.057 |
| Child never gets any vaccine | 719/9,998 (7.2%) | 290/2,342 (12%) | 26/847 (3.1%) | <0.001 | 349/3,400 (10%) | 40/2,176 (1.8%) | <0.001 | 13/733 (1.8%) | 1/500 (0.2%) | <0.001 |
| Vaccine not recommended for child's health history | 507/9,998 (5.1%) | 185/2,342 (7.9%) | 73/847 (8.6%) | 0.64 | 92/3,400 (2.7%) | 80/2,176 (3.7%) | 0.13 | 46/733 (6.3%) | 30/500 (6.1%) | 0.89 |
| Worried child will get COVID-19 from vaccine | 622/9,998 (6.2%) | 357/2,342 (15%) | 55/847 (6.4%) | <0.001 | 103/3,400 (3.0%) | 44/2,176 (2.0%) | 0.14 | 48/733 (6.5%) | 16/500 (3.3%) | 0.094 |
| Child is not at risk | 1,662/9,998 (17%) | 747/2,342 (32%) | 255/847 (30%) | 0.47 | 260/3,400 (7.6%) | 221/2,176 (10%) | 0.022 | 101/733 (14%) | 79/500 (16%) | 0.41 |
| Risk from vaccine greater than risk from COVID-19 | 3,601/9,998 (36%) | 1,743/2,342 (74%) | 613/847 (72%) | 0.4 | 333/3,400 (9.8%) | 165/2,176 (7.6%) | 0.031 | 443/733 (60%) | 303/500 (61%) | 0.97 |
| Child is afraid of needles | 479/9,998 (4.8%) | 176/2,342 (7.5%) | 38/847 (4.5%) | 0.017 | 97/3,400 (2.9%) | 89/2,176 (4.1%) | 0.08 | 40/733 (5.5%) | 37/500 (7.4%) | 0.34 |
| Vaccine is contrary to religious beliefs | 1,101/9,998 (11%) | 587/2,342 (25%) | 56/847 (6.6%) | <0.001 | 312/3,400 (9.2%) | 33/2,176 (1.5%) | <0.001 | 106/733 (14%) | 7/500 (1.4%) | <0.001 |
| Prefer to wait for herd immunity for protection | 1,360/9,998 (14%) | 645/2,342 (28%) | 251/847 (30%) | 0.39 | 171/3,400 (5.0%) | 137/2,176 (6.3%) | 0.14 | 92/733 (13%) | 63/500 (13%) | 0.98 |
| There are other people who should get it first | 223/9,998 (2.2%) | 76/2,342 (3.2%) | 25/847 (2.9%) | 0.68 | 30/3,400 (0.9%) | 59/2,176 (2.7%) | <0.001 | 17/733 (2.3%) | 17/500 (3.5%) | 0.41 |
| Other | 939/9,998 (9.4%) | 217/2,342 (9.3%) | 70/847 (8.2%) | 0.48 | 356/3,400 (10%) | 229/2,176 (11%) | 0.94 | 39/733 (5.3%) | 29/500 (5.7%) | 0.76 |
| Median Number of Reasons Given for Hesitancy | 3.0 (1.0 – 6.0) | 8.0 (6.0 – 10.0) | 7.0 (6.0 – 9.0) | <0.001 | 1.0 (1.0 – 2.0) | 1.0 (1.0 – 2.0) | 0.45 | 4.0 (3.0 – 5.0) | 4.0 (3.0 – 5.0) | 0.009 |

†chi-squared test with Rao & Scott's second-order correction; Wilcoxon rank-sum test for complex survey samples

*All Parents includes parents who are unvaccinated, partially vaccinated, fully vaccinated, and fully vaccinated and boosted

**Vaccinated parents includes parents who are partially vaccinated, fully vaccinated, or fully vaccinated and boosted
