# Supplementary material for: Parental compliance and reasons for COVID-19 Vaccination among American children
Source: PLOS Digit Health. 2023 Apr 12;2(4):e0000147. doi: 10.1371/journal.pdig.0000147 (PMC10096220; doi:10.1371/journal.pdig.0000147)
Supplement: S3 Table — (DOCX) [file pdig.0000147.s004.docx]

S3 Table. Characteristics of Parents with Uncertain or Inconsistent Preferences

|  | **Always Unsure or Sure (n=29,583)** | **Always Unsure (n=3,951)** | **Inconsistent Preferences Across Children (n=1,067)** |
| --- | --- | --- | --- |
|  | **n (%)** | **n (%)** | **n (%)** |
| Gender |  |  |  |
| Female | 15,304 (52%) | 2,114 (54%) | 606 (57%) |
| Male | 13,949 (47%) | 1,752 (44%) | 440 (41%) |
| Transgender or Nonbinary | 330 (1%) | 85 (2%) | 21 (2%) |
| Age |  |  |  |
| 18-29 years | 1,686 (6%) | 402 (10%) | 32 (3%) |
| 30-39 years | 8,018 (27%) | 1,421 (36%) | 372 (35%) |
| 40-49 years | 12,090 (41%) | 1,405 (36%) | 488 (46%) |
| 50-64 years | 6,872 (23%) | 614 (16%) | 148 (14%) |
| 65+ years | 917 (3%) | 109 (3%) | 27 (3%) |
| Household Income |  |  |  |
| Under $49,999 | 11,054 (37%) | 2,181 (55%) | 448 (42%) |
| $50,000-$99,999 | 8,384 (28%) | 1,056 (27%) | 332 (31%) |
| Over $100,000 | 10,146 (34%) | 714 (18%) | 287 (27%) |
| Race/Ethnicity |  |  |  |
| White, not Hispanic | 17,150 (58%) | 1,982 (50%) | 581 (54%) |
| Hispanic | 5,947 (20%) | 926 (23%) | 234 (22%) |
| Black | 3,640 (12%) | 740 (19%) | 148 (14%) |
| Asian | 1,677 (6%) | 131 (3%) | 48 (5%) |
| Other | 1,169 (4%) | 172 (4%) | 55 (5%) |
| Education |  |  |  |
| High School or Less | 11,294 (38%) | 2,005 (51%) | 430 (40%) |
| Some College | 9,075 (31%) | 1,342 (34%) | 372 (35%) |
| College Graduate | 9,215 (31%) | 603 (15%) | 265 (25%) |
| Employment Status |  |  |  |
| Employed | 24,388 (82%) | 2,929 (74%) | 875 (82%) |
| Unemployed | 5,195 (18%) | 1,022 (26%) | 192 (18%) |
| Health Insurance |  |  |  |
| Insured | 27,253 (92%) | 3,490 (88%) | 991 (93%) |
| Uninsured | 2,331 (8%) | 461 (12%) | 75 (7%) |
| Self Reported Health |  |  |  |
| Fair/Poor | 2,388 (8%) | 458 (12%) | 114 (11%) |
| Good | 7,588 (26%) | 1,190 (30%) | 305 (29%) |
| Very good | 11,194 (38%) | 1,344 (34%) | 384 (36%) |
| Excellent | 8,413 (28%) | 959 (24%) | 263 (25%) |
| Religious Status |  |  |  |
| Religious | 22,484 (76%) | 3,023 (77%) | 876 (82%) |
| Atheist/Agnostic | 7,099 (24%) | 928 (23%) | 191 (18%) |
| Parent COVID-19 Vaccination |  |  |  |
| Johnson | 1,970 (7%) | 290 (7%) | 88 (8%) |
| mRNA | 19,959 (67%) | 2,130 (54%) | 791 (74%) |
| Unvaccinated | 7,654 (26%) | 1,530 (39%) | 187 (18%) |
| Have Child Age 5 to 11 Years | 15,357 (52%) | 2,448 (62%) | 871 (82%) |
| Have Child Age 12 to 15 Years | 14,188 (48%) | 1,503 (38%) | 787 (74%) |
| Have Child Age 16 to 17 Years | 9,828 (33%) | 1,036 (26%) | 550 (52%) |
| Political Party Affiliation |  |  |  |
| Republican | 9,712 (33%) | 1,192 (30%) | 340 (32%) |
| Democrat | 9,538 (32%) | 1,012 (26%) | 302 (28%) |
| Independent | 10,334 (35%) | 1,747 (44%) | 424 (40%) |
| When Will the Pandemic End? |  |  |  |
| Already Over | 6,541 (22%) | 597 (15%) | 225 (21%) |
| Less than three months | 1,662 (6%) | 223 (6%) | 71 (7%) |
| Between three months and one year | 5,526 (19%) | 789 (20%) | 195 (18%) |
| More than one year | 15,854 (54%) | 2,342 (59%) | 576 (54%) |
| Flu Vaccine Since June 2021 | 13,935 (47%) | 1,306 (33%) | 437 (41%) |
