# Supplementary material for: Parental compliance and reasons for COVID-19 Vaccination among American children
Source: PLOS Digit Health. 2023 Apr 12;2(4):e0000147. doi: 10.1371/journal.pdig.0000147 (PMC10096220; doi:10.1371/journal.pdig.0000147)
Supplement: S4 Table — (DOCX) [file pdig.0000147.s005.docx]

S4 Table. Percent Willing to Vaccinate Children

| **Characteristic** | **All Parents†** | **Unvaccinated Parents** | **Fully Vaccinated Parents‡** |
| --- | --- | --- | --- |
| Gender |  |  |  |
| Female | 10,296/15,304 (67%) | 577/3,826 (15%) | 8,625/9,962 (87%) |
| Male | 9,119/13,949 (65%) | 577/3,694 (16%) | 7,768/9,102 (85%) |
| Transgender or Nonbinary | 171/330 (52%) | 25/135 (19%) | 135/168 (80%) |
| Age |  |  |  |
| 18-29 years | 827/1,686 (49%) | 178/744 (24%) | 548/748 (73%) |
| 30-39 years | 4,286/8,018 (53%) | 439/3,032 (14%) | 3,266/4,155 (79%) |
| 40-49 years | 8,334/12,090 (69%) | 413/2,764 (15%) | 7,185/8,274 (87%) |
| 50-64 years | 5,380/6,872 (78%) | 133/1,019 (13%) | 4,842/5,301 (91%) |
| 65+ years | 759/917 (83%) | 17/95 (18%) | 687/754 (91%) |
| Household Income |  |  |  |
| Under $49,999 | 6,919/11,054 (63%) | 808/3,744 (22%) | 5,053/5,897 (86%) |
| $50,000-$99,999 | 5,357/8,384 (64%) | 251/2,188 (11%) | 4,599/5,458 (84%) |
| Over $100,000 | 7,309/10,146 (72%) | 121/1,722 (7%) | 6,875/7,877 (87%) |
| Race/Ethnicity |  |  |  |
| White, not Hispanic | 10,311/17,150 (60%) | 573/5,108 (11%) | 9,043/10,889 (83%) |
| Hispanic | 724/1,169 (62%) | 46/336 (14%) | 582/695 (84%) |
| Black | 1,494/1,677 (89%) | 21/92 (23%) | 1,381/1,474 (94%) |
| Asian | 2,683/3,640 (74%) | 254/884 (29%) | 2,021/2,241 (90%) |
| Other | 4,374/5,947 (74%) | 285/1,235 (23%) | 3,501/3,934 (89%) |
| Education |  |  |  |
| High School or Less | 6,760/11,294 (60%) | 784/4,031 (19%) | 4,942/5,849 (84%) |
| Some College | 5,742/9,075 (63%) | 297/2,405 (12%) | 4,893/5,874 (83%) |
| College Graduate | 7,085/9,215 (77%) | 98/1,219 (8%) | 6,693/7,509 (89%) |
| Employment Status |  |  |  |
| Employed | 16,141/24,388 (66%) | 761/5,904 (13%) | 13,932/16,346 (85%) |
| Unemployed | 3,445/5,195 (66%) | 418/1,750 (24%) | 2,595/2,886 (90%) |
| Health Insurance |  |  |  |
| Insured | 18,335/27,253 (67%) | 955/6,558 (15%) | 15,699/18,269 (86%) |
| Uninsured | 1,251/2,331 (54%) | 225/1,096 (21%) | 828/963 (86%) |
| Self Reported Health |  |  |  |
| Fair/Poor | 1,583/2,388 (66%) | 148/663 (22%) | 1,232/1,445 (85%) |
| Good | 5,085/7,588 (67%) | 309/1,926 (16%) | 4,266/4,942 (86%) |
| Very good | 7,566/11,194 (68%) | 354/2,653 (13%) | 6,575/7,610 (86%) |
| Excellent | 5,352/8,413 (64%) | 369/2,413 (15%) | 4,453/5,235 (85%) |
| Religious Status |  |  |  |
| Religious | 14,614/22,484 (65%) | 865/5,870 (15%) | 12,211/14,412 (85%) |
| Atheist/Agnostic | 4,972/7,099 (70%) | 314/1,784 (18%) | 4,316/4,820 (90%) |
| Have Child Age 5 to 11 Years |  |  |  |
| No | 10,443/14,226 (73%) | 589/3,197 (18%) | 8,788/9,665 (91%) |
| Yes | 9,143/15,357 (60%) | 591/4,457 (13%) | 7,739/9,567 (81%) |
| Have Child Age 12 to 15 Years |  |  |  |
| No | 10,122/15,395 (66%) | 671/3,906 (17%) | 8,530/10,087 (85%) |
| Yes | 9,464/14,188 (67%) | 509/3,748 (14%) | 7,997/9,145 (87%) |
| Have Child Age 16 to 17 Years |  |  |  |
| No | 12,550/19,756 (64%) | 738/5,229 (14%) | 10,585/12,684 (83%) |
| Yes | 7,036/9,828 (72%) | 442/2,425 (18%) | 5,943/6,548 (91%) |
| Political Party Affiliation |  |  |  |
| Republican | 4,946/9,712 (51%) | 306/3,340 (9%) | 4,103/5,444 (75%) |
| Democrat | 8,307/9,538 (87%) | 368/1,040 (35%) | 7,199/7,602 (95%) |
| Independent | 6,333/10,334 (61%) | 505/3,274 (15%) | 5,225/6,186 (84%) |
| Parent Vaccination Status |  |  |  |
| Unvaccinated | 1,180/7,654 (15%) | 1,180/7,654 (15%) | 0/0 (NA%) |
| Partially Vaccinated | 1,879/2,697 (70%) | 0/0 (NA%) | 0/0 (NA%) |
| Fully Vaccinated | 7,490/9,713 (77%) | 0/0 (NA%) | 7,490/9,713 (77%) |
| Fully Vaccinated and Boosted | 9,037/9,519 (95%) | 0/0 (NA%) | 9,037/9,519 (95%) |
| When Will the Pandemic End? |  |  |  |
| Already Over | 195/3,371 (6%) | 195/3,371 (6%) | 1,654/2,681 (62%) |
| Less than three months | 83/395 (21%) | 83/395 (21%) | 929/1,116 (83%) |
| Between three months and one year | 223/988 (23%) | 223/988 (23%) | 3,705/4,059 (91%) |
| More than one year | 679/2,901 (23%) | 679/2,901 (23%) | 10,239/11,376 (90%) |
| Flu Vaccine Since June 2021 | 219/820 (27%) | 219/820 (27%) | 10,778/11,998 (90%) |
| Parent Vaccination Type |  |  |  |
| Johnson | 1,409/1,970 (72%) | 0/0 (NA%) | 1,189/1,607 (74%) |
| mRNA | 16,997/19,959 (85%) | 0/0 (NA%) | 15,338/17,625 (87%) |
| Unvaccinated | 1,180/7,654 (15%) | 1,180/7,654 (15%) | 0/0 (NA%) |

†All Parents includes parents who are unvaccinated, partially vaccinated, fully vaccinated, and fully vaccinated and boosted

‡Fully vaccinated includes parents who are either fully vaccinated or fully vaccinated and boosted and excludes those partially vaccinated
