# Supplementary material for: Parental compliance and reasons for COVID-19 Vaccination among American children
Source: PLOS Digit Health. 2023 Apr 12;2(4):e0000147. doi: 10.1371/journal.pdig.0000147 (PMC10096220; doi:10.1371/journal.pdig.0000147)
Supplement: S5 Table — (DOCX) [file pdig.0000147.s006.docx]

S5 Table. Multivariate Relationship between Parent Characteristics and Willingness to Vaccinate Children, Varying Reference Level for Parent Vaccination Status†

| All Parents | | | | |
| --- | --- | --- | --- | --- |
|  | **Unvaccinated Reference** | **Partially Vaccinated Reference** | **Fully Vaccinated Reference** | **Fully Vaccinated and Boosted Reference** |
|  | **Odds Ratio (95% confidence interval)** | **Odds Ratio (95% confidence interval)** | **Odds Ratio (95% confidence interval)** | **Odds Ratio (95% confidence interval)** |
| Parent Vaccination Status |  |  |  |  |
| Unvaccinated | — | 0.08 (0.08, 0.09)*** | 0.05 (0.05, 0.05)*** | 0.01 (0.01, 0.01)*** |
| Partially Vaccinated | 11.9 (10.6, 13.3)*** | — | 0.60 (0.54, 0.66)*** | 0.11 (0.10, 0.13)*** |
| Fully Vaccinated | 19.9 (18.2, 21.7)*** | 1.67 (1.51, 1.86)*** | — | 0.19 (0.17, 0.21)*** |
| Fully Vaccinated and Boosted | 106 (93.9, 120)*** | 8.94 (7.83, 10.2)*** | 5.34 (4.80, 5.96)*** | — |

*p<.05; **p<.01; ***p<.001

†Table 2 displays full multivariate regression results
