# Supplementary material for: Parental compliance and reasons for COVID-19 Vaccination among American children
Source: PLOS Digit Health. 2023 Apr 12;2(4):e0000147. doi: 10.1371/journal.pdig.0000147 (PMC10096220; doi:10.1371/journal.pdig.0000147)
Supplement: S6 Table — (DOCX) [file pdig.0000147.s007.docx]

S6 Table. Multivariate Results, Stratification by Age

|  | **Willingness to Vaccinate Children** | |
| --- | --- | --- |
|  | **>= 50 years** | **< 50 years** |
|  | **Odds Ratio (95% confidence interval)** | **Odds Ratio (95% confidence interval)** |
| Gender |  |  |
| Female | — | — |
| Male | 1.00 (0.85, 1.19) | 1.23 (1.13, 1.33)*** |
| Transgender or Nonbinary | 0.30 (0.14, 0.64)** | 0.80 (0.57, 1.11) |
| Household Income |  |  |
| Under $49,999 | — | — |
| $50,000-$99,999 | 0.95 (0.75, 1.20) | 0.74 (0.67, 0.82)*** |
| Over $100,000 | 0.74 (0.57, 0.94)* | 0.77 (0.69, 0.87)*** |
| Race/Ethnicity |  |  |
| White, not Hispanic | — | — |
| Hispanic | 1.48 (1.18, 1.86)*** | 1.88 (1.70, 2.08)*** |
| Black | 2.91 (2.03, 4.25)*** | 1.48 (1.30, 1.68)*** |
| Asian | 3.54 (2.27, 5.73)*** | 3.09 (2.53, 3.80)*** |
| Other | 0.61 (0.42, 0.88)** | 1.31 (1.08, 1.59)** |
| Education |  |  |
| High School or Less | — | — |
| Some College | 0.67 (0.54, 0.83)*** | 0.85 (0.77, 0.93)*** |
| College Graduate | 0.85 (0.68, 1.06) | 1.05 (0.94, 1.17) |
| Employment Status |  |  |
| Employed | — | — |
| Unemployed | 1.42 (1.13, 1.78)** | 1.58 (1.42, 1.76)*** |
| Health Insurance |  |  |
| Insured | — | — |
| Uninsured | 0.63 (0.44, 0.92)* | 1.32 (1.15, 1.50)*** |
| Self Reported Health |  |  |
| Fair/Poor | — | — |
| Good | 0.52 (0.36, 0.75)*** | 1.17 (1.01, 1.36)* |
| Very good | 0.61 (0.42, 0.87)** | 1.13 (0.98, 1.31) |
| Excellent | 0.57 (0.39, 0.83)** | 1.05 (0.91, 1.22) |
| Religious Status |  |  |
| Religious | — | — |
| Atheist/Agnostic | 1.42 (1.13, 1.78)** | 1.32 (1.20, 1.44)*** |
| Have Child Age 5 to 11 Years |  |  |
| No | — | — |
| Yes | 0.48 (0.39, 0.60)*** | 0.50 (0.45, 0.54)*** |
| Have Child Age 12 to 15 Years |  |  |
| No | — | — |
| Yes | 1.07 (0.88, 1.30) | 1.06 (0.98, 1.15) |
| Have Child Age 16 to 17 Years |  |  |
| No | — | — |
| Yes | 1.47 (1.19, 1.81)*** | 1.36 (1.24, 1.50)*** |
| Political Party Affiliation |  |  |
| Republican | — | — |
| Democrat | 6.59 (5.13, 8.54)*** | 3.88 (3.49, 4.33)*** |
| Independent | 1.41 (1.18, 1.69)*** | 1.58 (1.45, 1.73)*** |
| Parent Vaccination Status |  |  |
| Unvaccinated | — | — |
| Partially Vaccinated | 15.5 (11.8, 20.5)*** | 11.5 (10.2, 13.1)*** |
| Fully Vaccinated | 32.2 (25.7, 40.5)*** | 18.2 (16.5, 20.0)*** |
| Fully Vaccinated and Boosted | 162 (124, 213)*** | 98.5 (85.6, 114)*** |

*p<.05; **p<.01; ***p<.001
