# Supplementary material for: Parental compliance and reasons for COVID-19 Vaccination among American children
Source: PLOS Digit Health. 2023 Apr 12;2(4):e0000147. doi: 10.1371/journal.pdig.0000147 (PMC10096220; doi:10.1371/journal.pdig.0000147)
Supplement: S7 Table — (DOCX) [file pdig.0000147.s008.docx]

S7 Table. Multivariate Results, Stratification by Education

|  | **Willingness to Vaccinate Children** | |
| --- | --- | --- |
|  | **College Graduate** | **Not College Graduate** |
|  | **Odds Ratio (95% confidence interval)** | **Odds Ratio (95% confidence interval)** |
| Gender |  |  |
| Female | — | — |
| Male | 1.00 (0.86, 1.17) | 1.24 (1.14, 1.35)*** |
| Transgender or Nonbinary | 0.38 (0.21, 0.68)** | 0.95 (0.67, 1.36) |
| Age |  |  |
| 18-29 years | — | — |
| 30-39 years | 1.47 (0.88, 2.43) | 0.96 (0.82, 1.11) |
| 40-49 years | 2.20 (1.33, 3.60)** | 1.23 (1.05, 1.43)* |
| 50-64 years | 2.45 (1.46, 4.07)*** | 1.45 (1.21, 1.73)*** |
| 65+ years | 2.96 (1.51, 5.84)** | 1.14 (0.84, 1.55) |
| Household Income |  |  |
| Under $49,999 | — | — |
| $50,000-$99,999 | 0.73 (0.56, 0.96)* | 0.74 (0.67, 0.82)*** |
| Over $100,000 | 0.62 (0.48, 0.81)*** | 0.70 (0.62, 0.79)*** |
| Race/Ethnicity |  |  |
| White, not Hispanic | — | — |
| Hispanic | 1.57 (1.24, 2.00)*** | 1.96 (1.77, 2.17)*** |
| Black | 1.72 (1.26, 2.36)*** | 1.63 (1.44, 1.86)*** |
| Asian | 4.26 (3.15, 5.86)*** | 2.48 (1.96, 3.17)*** |
| Other | 0.81 (0.58, 1.14) | 1.29 (1.06, 1.57)* |
| Employment Status |  |  |
| Employed | — | — |
| Unemployed | 1.35 (1.02, 1.79)* | 1.61 (1.45, 1.78)*** |
| Health Insurance |  |  |
| Insured | — | — |
| Uninsured | 0.78 (0.50, 1.21) | 1.26 (1.11, 1.44)*** |
| Self Reported Health |  |  |
| Fair/Poor | — | — |
| Good | 1.22 (0.85, 1.74) | 1.01 (0.87, 1.17) |
| Very good | 1.24 (0.87, 1.74) | 0.99 (0.86, 1.15) |
| Excellent | 1.07 (0.75, 1.51) | 0.98 (0.84, 1.14) |
| Religious Status |  |  |
| Religious | — | — |
| Atheist/Agnostic | 1.41 (1.17, 1.72)*** | 1.30 (1.18, 1.43)*** |
| Have Child Age 5 to 11 Years |  |  |
| No | — | — |
| Yes | 0.48 (0.39, 0.57)*** | 0.52 (0.48, 0.58)*** |
| Have Child Age 12 to 15 Years |  |  |
| No | — | — |
| Yes | 1.00 (0.86, 1.18) | 1.07 (0.98, 1.17) |
| Have Child Age 16 to 17 Years |  |  |
| No | — | — |
| Yes | 1.45 (1.20, 1.75)*** | 1.34 (1.21, 1.48)*** |
| Political Party Affiliation |  |  |
| Republican | — | — |
| Democrat | 7.68 (6.15, 9.66)*** | 3.52 (3.15, 3.94)*** |
| Independent | 1.52 (1.29, 1.78)*** | 1.56 (1.43, 1.71)*** |
| Parent Vaccination Status |  |  |
| Unvaccinated | — | — |
| Partially Vaccinated | 17.1 (12.7, 23.2)*** | 11.6 (10.2, 13.1)*** |
| Fully Vaccinated | 41.9 (32.9, 53.9)*** | 17.1 (15.5, 18.9)*** |
| Fully Vaccinated and Boosted | 279 (212, 373)*** | 75.8 (65.8, 87.6)*** |

*p<.05; **p<.01; ***p<.001
