# Supplementary material for: Parental compliance and reasons for COVID-19 Vaccination among American children
Source: PLOS Digit Health. 2023 Apr 12;2(4):e0000147. doi: 10.1371/journal.pdig.0000147 (PMC10096220; doi:10.1371/journal.pdig.0000147)
Supplement: S8 Table — (DOCX) [file pdig.0000147.s009.docx]

S8 Table. Multivariate Results, Stratification by Income

|  | **Willingness to Vaccinate Children** | |
| --- | --- | --- |
|  | **Income > $100,000** | **Income <= $100,000** |
|  | **Odds Ratio (95% confidence interval)** | **Odds Ratio (95% confidence interval)** |
| Gender |  |  |
| Female | — | — |
| Male | 0.98 (0.85, 1.13) | 1.18 (1.09, 1.29)*** |
| Transgender or Nonbinary | 0.37 (0.21, 0.64)*** | 0.90 (0.63, 1.29) |
| Age |  |  |
| 18-29 years | — | — |
| 30-39 years | 0.74 (0.44, 1.23) | 1.00 (0.86, 1.16) |
| 40-49 years | 0.83 (0.50, 1.37) | 1.33 (1.14, 1.56)*** |
| 50-64 years | 0.93 (0.55, 1.56) | 1.55 (1.30, 1.86)*** |
| 65+ years | 0.68 (0.35, 1.34) | 1.55 (1.15, 2.10)** |
| Race/Ethnicity |  |  |
| White, not Hispanic | — | — |
| Hispanic | 1.79 (1.45, 2.21)*** | 1.92 (1.73, 2.13)*** |
| Black | 1.62 (1.17, 2.26)** | 1.72 (1.51, 1.95)*** |
| Asian | 3.08 (2.28, 4.21)*** | 3.17 (2.51, 4.05)*** |
| Other | 0.51 (0.37, 0.72)*** | 1.52 (1.25, 1.85)*** |
| Education |  |  |
| High School or Less | — | — |
| Some College | 0.60 (0.49, 0.74)*** | 0.83 (0.76, 0.91)*** |
| College Graduate | 0.76 (0.62, 0.92)** | 0.95 (0.84, 1.08) |
| Employment Status |  |  |
| Employed | — | — |
| Unemployed | 1.62 (1.21, 2.18)** | 1.61 (1.45, 1.78)*** |
| Health Insurance |  |  |
| Insured | — | — |
| Uninsured | 1.07 (0.68, 1.71) | 1.26 (1.11, 1.43)*** |
| Self Reported Health |  |  |
| Fair/Poor | — | — |
| Good | 0.98 (0.68, 1.40) | 1.02 (0.88, 1.19) |
| Very good | 0.99 (0.69, 1.39) | 0.99 (0.86, 1.15) |
| Excellent | 0.86 (0.60, 1.21) | 1.00 (0.86, 1.16) |
| Religious Status |  |  |
| Religious | — | — |
| Atheist/Agnostic | 1.74 (1.46, 2.07)*** | 1.21 (1.10, 1.34)*** |
| Have Child Age 5 to 11 Years |  |  |
| No | — | — |
| Yes | 0.41 (0.35, 0.49)*** | 0.55 (0.50, 0.61)*** |
| Have Child Age 12 to 15 Years |  |  |
| No | — | — |
| Yes | 1.07 (0.93, 1.24) | 1.05 (0.96, 1.15) |
| Have Child Age 16 to 17 Years |  |  |
| No | — | — |
| Yes | 1.23 (1.04, 1.45)* | 1.40 (1.27, 1.56)*** |
| Political Party Affiliation |  |  |
| Republican | — | — |
| Democrat | 8.12 (6.46, 10.3)*** | 3.57 (3.19, 4.00)*** |
| Independent | 1.38 (1.19, 1.59)*** | 1.66 (1.51, 1.83)*** |
| Parent Vaccination Status |  |  |
| Unvaccinated | — | — |
| Partially Vaccinated | 17.3 (13.2, 22.8)*** | 11.1 (9.77, 12.6)*** |
| Fully Vaccinated | 38.1 (30.7, 47.7)*** | 16.7 (15.1, 18.4)*** |
| Fully Vaccinated and Boosted | 275 (214, 357)*** | 67.0 (57.9, 77.7)*** |

*p<.05; **p<.01; ***p<.001
