# Supplementary material for: Parental compliance and reasons for COVID-19 Vaccination among American children
Source: PLOS Digit Health. 2023 Apr 12;2(4):e0000147. doi: 10.1371/journal.pdig.0000147 (PMC10096220; doi:10.1371/journal.pdig.0000147)
Supplement: S9 Table — (DOCX) [file pdig.0000147.s010.docx]

S9 Table. Multivariate Results, Stratification by Political Party

|  | **Willingness to Vaccinate Children** | |
| --- | --- | --- |
|  |  |  |
|  | **Odds Ratio (95% confidence interval)** | **Odds Ratio (95% confidence interval)** |
| Gender |  |  |
| Female | — | — |
| Male | 1.24 (1.05, 1.46)* | 1.15 (1.06, 1.25)*** |
| Transgender or Nonbinary | 0.39 (0.19, 0.81)* | 0.82 (0.59, 1.16) |
| Age |  |  |
| 18-29 years | — | — |
| 30-39 years | 1.03 (0.78, 1.37) | 1.01 (0.85, 1.19) |
| 40-49 years | 1.70 (1.26, 2.28)*** | 1.25 (1.05, 1.48)* |
| 50-64 years | 3.10 (2.18, 4.42)*** | 1.32 (1.09, 1.59)** |
| 65+ years | 2.11 (1.24, 3.77)** | 1.19 (0.87, 1.62) |
| Household Income |  |  |
| Under $49,999 | — | — |
| $50,000-$99,999 | 0.90 (0.75, 1.09) | 0.69 (0.62, 0.76)*** |
| Over $100,000 | 1.17 (0.91, 1.51) | 0.63 (0.56, 0.71)*** |
| Race/Ethnicity |  |  |
| White, not Hispanic | — | — |
| Hispanic | 1.10 (0.90, 1.36) | 2.21 (1.99, 2.45)*** |
| Black | 1.00 (0.83, 1.22) | 2.50 (2.14, 2.94)*** |
| Asian | 2.05 (1.35, 3.19)** | 3.36 (2.74, 4.14)*** |
| Other | 1.12 (0.76, 1.67) | 1.19 (0.98, 1.44) |
| Education |  |  |
| High School or Less | — | — |
| Some College | 1.12 (0.94, 1.34) | 0.72 (0.66, 0.79)*** |
| College Graduate | 1.35 (1.07, 1.72)* | 0.88 (0.79, 0.99)* |
| Employment Status |  |  |
| Employed | — | — |
| Unemployed | 1.29 (1.06, 1.56)** | 1.69 (1.51, 1.90)*** |
| Health Insurance |  |  |
| Insured | — | — |
| Uninsured | 1.21 (0.92, 1.59) | 1.23 (1.07, 1.42)** |
| Self Reported Health |  |  |
| Fair/Poor | — | — |
| Good | 1.16 (0.89, 1.52) | 1.02 (0.87, 1.19) |
| Very good | 1.30 (1.00, 1.69)* | 0.98 (0.84, 1.15) |
| Excellent | 1.15 (0.88, 1.50) | 0.93 (0.79, 1.08) |
| Religious Status |  |  |
| Religious | — | — |
| Atheist/Agnostic | 1.63 (1.34, 1.98)*** | 1.36 (1.24, 1.50)*** |
| Have Child Age 5 to 11 Years |  |  |
| No | — | — |
| Yes | 0.73 (0.60, 0.88)** | 0.48 (0.43, 0.52)*** |
| Have Child Age 12 to 15 Years |  |  |
| No | — | — |
| Yes | 1.36 (1.15, 1.61)*** | 1.00 (0.92, 1.09) |
| Have Child Age 16 to 17 Years |  |  |
| No | — | — |
| Yes | 1.25 (1.02, 1.52)* | 1.38 (1.25, 1.52)*** |
| Parent Vaccination Status |  |  |
| Unvaccinated | — | — |
| Partially Vaccinated | 7.76 (6.19, 9.78)*** | 13.2 (11.6, 15.0)*** |
| Fully Vaccinated | 13.8 (11.4, 16.7)*** | 21.3 (19.3, 23.7)*** |
| Fully Vaccinated and Boosted | 61.0 (46.6, 80.8)*** | 122 (106, 140)*** |

*p<.05; **p<.01; ***p<.001
