# Supplementary material for: Parental compliance and reasons for COVID-19 Vaccination among American children
Source: PLOS Digit Health. 2023 Apr 12;2(4):e0000147. doi: 10.1371/journal.pdig.0000147 (PMC10096220; doi:10.1371/journal.pdig.0000147)
Supplement: S10 Table — (DOCX) [file pdig.0000147.s011.docx]

S10 Table. Reasons for Refusal by Child Age

|  | **Age 5-11 (N=6,214)** | **Age 12-15 (N = 4,725)** | **Age 16-17 (N = 2,792)** |
| --- | --- | --- | --- |
| **Reason** |  |  |  |
| Child received flu vaccine and is protected | 23/6,214 (0%) | 24/4,725 (1%) | 23/2,792 (1%) |
| Vaccine too new | 2,706/6,214 (44%) | 2,136/4,725 (45%) | 1,221/2,792 (44%) |
| Child too young | 1,746/6,214 (28%) | 941/4,725 (20%) | 421/2,792 (15%) |
| Let child decide when older | 1,143/6,214 (18%) | 994/4,725 (21%) | 748/2,792 (27%) |
| Side Effects | 2,881/6,214 (46%) | 2,326/4,725 (49%) | 1,337/2,792 (48%) |
| COVID-19 threat exaggerated | 1,413/6,214 (23%) | 1,256/4,725 (27%) | 785/2,792 (28%) |
| Lack trust in government | 2,403/6,214 (39%) | 1,985/4,725 (42%) | 1,206/2,792 (43%) |
| Lack trust in scientists | 2,034/6,214 (33%) | 1,693/4,725 (36%) | 1,079/2,792 (39%) |
| Vaccine development too political | 2,113/6,214 (34%) | 1,840/4,725 (39%) | 1,098/2,792 (39%) |
| Child already had COVID-19 | 1,469/6,214 (24%) | 1,311/4,725 (28%) | 851/2,792 (30%) |
| Child never gets any vaccine | 453/6,214 (7%) | 368/4,725 (8%) | 207/2,792 (7%) |
| Vaccine not recommended for child's health history | 292/6,214 (5%) | 261/4,725 (6%) | 167/2,792 (6%) |
| Worried child will get COVID-19 from vaccine | 399/6,214 (6%) | 303/4,725 (6%) | 156/2,792 (6%) |
| Child is not at risk | 1,025/6,214 (16%) | 848/4,725 (18%) | 467/2,792 (17%) |
| Risk from vaccine greater than risk from COVID-19 | 2,106/6,214 (34%) | 1,909/4,725 (40%) | 1,100/2,792 (39%) |
| Child is afraid of needles | 350/6,214 (6%) | 185/4,725 (4%) | 104/2,792 (4%) |
| Vaccine is contrary to religious beliefs | 628/6,214 (10%) | 591/4,725 (13%) | 387/2,792 (14%) |
| Prefer to wait for herd immunity for protection | 780/6,214 (13%) | 666/4,725 (14%) | 463/2,792 (17%) |
| There are other people who should get it first | 134/6,214 (2%) | 97/4,725 (2%) | 63/2,792 (2%) |
| Other | 524/6,214 (8%) | 460/4,725 (10%) | 322/2,792 (12%) |

We see that across child age, parents list reasons with very similar rates. Note that a parent can appear in multiple columns if they have multiple children across age categories.
