# Supplementary material for: Parental compliance and reasons for COVID-19 Vaccination among American children
Source: PLOS Digit Health. 2023 Apr 12;2(4):e0000147. doi: 10.1371/journal.pdig.0000147 (PMC10096220; doi:10.1371/journal.pdig.0000147)
Supplement: Attachment — Submitted filename: PLOS DH Response to Reviewers V3.pdf [file pdig.0000147.s012.pdf]

Dear Dr. Tizzoni,

Thank you for the opportunity to revise our manuscript. The reviewers brought up many important points and we were glad to have had the opportunity to improve the manuscript. We have responded below to your and the reviewer's comments.

We hope that this revision brings our work to the standards of *PLOS Digital Health*, and we would be happy to consider additional revisions if necessary. Thank you for your continued interest in our research. Please let us know if we can provide any further details.

Sincerely,

Neil Sehgal, AB
Harvard School of Engineering and Applied Sciences & Boston Children's Hospital
Boston, MA 02134
neil_sehgal@g.harvard.edu

Reviewer #1:

This paper aim to characterize factors of parental vaccination hesitancy among their children using data from large-scale US surveys. They found that the willingness to vaccinate a child for COVID-19 was strongly associated with parental vaccination status. In particular, parental age and education is positively associated with willingness for vaccinated parents. Last, they characterized three groups of hesitant parents based on their reasons for vaccinate hesitancy. empirical data provides insights on the role of complex social processes in driving spatial heterogeneity in vaccine hesitancy. This observational study provides interesting insights on factors driving heterogeneity in vaccine hesitancy, but, at this stage, it cannot be published.

Thank you for your helpful comments.

I have a few recommendations for improvement or clarification.

Major Comments

1) In the paper, you discussed as in previous studies the sample of survey participants were corrected to reflect the US adult population in demographic composition and political beliefs. After the filtering, how did you re-weight the sample? I think this sentence "After exclusion, the mean survey weight of participants was .98, leading to a weighted sample of 29,583" needs clarifications, and the manuscript would benefit of a panel with figures showing statistics on respondents before and after filtering.

Thank you for this comment. Weights were generated from all 217,023 respondents to target US census demographics. Weights totaled <1 because the sample that met the inclusion criteria were not fully representative of the overall study population. We have added clarification in the text (page 2, lines 110-114) and have added a subject selection flowchart to the appendix (Appendix Figure 1) to better explain the filtering process.

2) Di you test cross-correlation among indicators used in the analysis, such as between "less education" and "a lack of health insurance"? I expected these are highly correlated, and I think the manuscript will benefit about more insight on the interconnection of some factors.

Thank you for the comment. VIF analysis suggests multicollinearity has limited effect on our regressions. In all four multivariate regressions, squared scaled GVIFs are <1.5. We now specify this in the text (page 4, lines 177-178).

Minor Comments

1) Please add in this sentence of the abstract 'Among hesitant parents, parental vaccination status was inversely associated with reported lack of trust in government (p<.001) and scientists (p<.001)', the values of the negative significant correlation.

Thank you for the suggestion, We have updated the abstract to be more specific (lines 20-21).

2) please take in consideration to cite this work on Spatial clustering in vaccination hesitancy: *https://journals.plos.org/ploscompbiol/article?id=10.1371/journal.pcbi.1010437*

Thank you for suggesting this paper. We now cite this work in our discussion (page 6, lines 279-283).

Reviewer #2:

This study explores the reasons for vaccine hesitancy in parents regarding Covid-19 vaccination among their children. This work relies on data from cross-sectional surveys representing a robust sample of the US population. The paper is well-written and easy to follow and the statistical analysis is technically sound.

Thank you for your helpful comments.

I have a few suggestions regarding the method's section:

1. I would recommend writing the equation for the multivariate logistic regression as log(P(y=1)/P(y=0))=\alpha+\beta... rather than Y=....

Thank you for the suggestion. We have adjusted the equation (page 2, line 119).

2. Can the authors provide more details about the weights W and how are they implemented?

Thank you for the suggestion. In a joint Boston Children's Hospital - US CDC analysis comparing the weighted and unweighted populations, we found weighted populations closely approximated the demographic composition of US adults (http://dx.doi.org/10.15585/mmwr.mm7113e1, Supplementary Table 1). We have additionally added more clarification to the text (page 2, lines 110-114) and a subject selection flow chart (Appendix Figure 1).

3. I appreciate that the authors shared the data to perform the analysis, and it would be useful to provide the code on github too.

Thank you for the suggestion. We have shared the code (page 7, line 315).

4. Third paragraph in the Discussion section, line 2: oft should be often?

Thank you for this suggestion. We have changed the word usage (page 6, line 252).

Reviewer #3:

The study and manuscript examine reasons related to parent/caregiver COVID-19 vaccination for their children. Data from cross-sectional surveys undertaken between January 1 and May 9, 2022, were pooled to create a sample of 30,174 U.S. parents with at least one child of COVID-19 vaccine eligible age (5-17 years old). Respondents answered questions about demographics, pandemic-related behavior, and COVID-19 self and child vaccination status. Major findings included: fully vaccinated and unvaccinated parents reported concordant vaccination preferences for their eligible child; high parent age and education were positively associated with willingness to vaccinate their children; and cluster analysis revealed three groups of hesitant parents.

Thank you for your helpful comments.

Comments and suggestions to the authors:

1) The use of the phrase "parental preferences" in the title is confusing given the focus of this study was on identifying factors, primarily demographic, related to parents' compliance with COVID-19 vaccination recommendations for their 5 to 17 year-old children. The word "preferences" does not appear in the Introduction or Survey Question areas of the manuscript. It would be better to frame this as an effort to identify factors associated with parent compliance.

Thank you for this suggestion. We have updated the title and the text throughout to focus on compliance.

2) While the Abstract and Introduction mention the word hesitancy, the term is never defined. The lack of a definition is particularly problematic given a) it does not appear that any survey questions sought to measure hesitancy (e.g., parent doubt or reluctance regarding COVID-19 vaccines or vaccination) and b) the analyses did not include parents who indicated their were "unsure" about having their child receive a COVID-19 vaccine/vaccination. It seems that parents who are "unsure" best reflect actual hesitancy (i.e., they are currently reluctant), whereas parents who have declined or do not intend to have their children receive a COVID-19 are more accurately characterized as "decliners" or "refusers." It is pretty clear these parents have made a clear decision with respect to vaccination and thus are not actually hesitant. This research and manuscript would be much more helpful if it accurately characterized parents and included "unsure" parents in the analyses.

Thank you for this comment. We have updated the text throughout to give the paper a clearer focus on firm willingness/refusal to vaccinate, as opposed to hesitancy.

3) It is worrisome that in the Introduction the authors state that prior research has been "limited by potentially non-representative samples," when, as indicated in the Discussion, one of the limitations of this study is that uses a "convenience sample" . . . "that may not be representative of the public as a whole." In addition, while the authors state in the Discussion that "previous research has suggested that the survey is representative of the larger population [25]," the study cited (i.e., 25) a) states upfront that the data collection method is "an ongoing prospective, nonprobability-based cross-sectional online survey" (which is a better characterization) and b) mentions as a first limitation "the survey uses a nonprobability-based sample and the results might not be generalizable to the U.S. population."

Thank you for your comment. In the cited study, a joint Boston Children's Hospital - US CDC analysis comparing the weighted and unweighted populations, we found weighted populations to be highly representative of US adults in demographic categories we targeted (http://dx.doi.org/10.15585/mmwr.mm7113e1, Supplementary Table 1). However, it must be acknowledged the sample may not be representative for non-targeted demographics such as subgroups without internet access. We have updated the discussion to highlight this (page 5, lines 236-238 and page 6 lines 268-271).

4) The relatively short Discussion devotes an entire paragraph to the WHO 3 Cs framework, but that framework appears to be applied post hoc (i.e., it was not the basis for survey questions or measures). At a minimum, if the 3 Cs framework is to be used it should have been introduced in the Introduction and set up in the Methods section.

Thank you for this suggestion. We have rephrased this paragraph to focus on contextualizing the clustering results without explicitly invoking the WHO 3 Cs framework post hoc (page 6, lines 251-263).

5) It is stated in the Discussion that the results suggest "better tailoring" of messages to increase vaccination rates among children but no specifics or examples are provided. As such, this guidance has almost no value.

Thank you for the comment. We have removed the sentence and modified phrasing in the concluding paragraph that the work presented here can be used to inform this work: "The results presented here can inform the further work needed to tailor child vaccination efforts to subgroups of parents who are more reluctant" (Page 6, line 286-288).

6) While the tables provide much information, the information in the tables is difficult to interpret. It would be helpful if Table 2 included the percentages as well as the odds ratios and indicated which were statistically significant (e.g., via bolding). Tables 3 does not indicate which results are statistically significant within clusters or between clusters. The same issues are true for the appendix tables.

Thank you for the comment. We have included an appendix table (Appendix Table 4) to display the responses on willingness to vaccinate. We have updated Table 2, Appendix Table 1, to include asterisks for displaying statistical significance. Table 3 and Appendix Table 2 additionally now display statistical significance between clusters.

Reviewer #4:

In this paper, dr Sehgal and colleagues investigate the willingness to vaccinate children from a survey of US self-reported parents. They found that parent vaccination, as well as a many other parental characteristics, is associated with an increase willingness to vaccinate their children. I would be grateful if the authors could address my questions and comments regarding their approach.

Thank you for your helpful comments.

1) I suggest author to not define parent vaccination as a determinant for the willingness, more likely, the set of characteristics that increase the likelihood of being vaccinated in parents also increase the likelihood of being willing to vaccinate the children. I would reframe the discussion and the analysis to better investigate/highlight this aspect

Thank you for this suggestion. We now include an additional regression analysis excluding parent vaccination status. In this model not using parent vaccination status as a determinant of willingness, we find that major predictors of parents' preferences for child vaccination still include Democrat political affiliation (OR: 6.03, CI: 5.57-6.53) and Asian race (OR: 5.15, CI: 4.37-6.09). In addition, we find that high income and having health insurance are independently positively associated with willingness, whereas we observed negative associations in the model including parental vaccination

status. We updated the text (page 3, lines 133-136 and page 4, line 182-187) and Table 2 to include this result.

2) Given the broad range of epidemiological condition worldwide, I would suggest authors to make explicit both in the title and in the background that the data are from only one country, the United States. Moreover, I would suggest authors to better frame their findings with respect to vaccination coverage for children in other countries, is it the lower rate only a US problem?

Thank you for this suggestion. We have clarified the title and in the introduction (page 1, line 72).

3) Method: "Each day, over 2 million individuals complete online surveys" where? In the US? Was the survey anonymous?

Thank you for this comment. Over 2 million individuals globally complete surveys on SurveyMonkey each day. We invited US SurveyMonkey respondents to anonymously participate in our survey. We have clarified in the text (page 2, lines 83-86).

Survey Questions.
4) how have authors addressed parents who already have vaccinated their children? Likewise, why restricting the analysis to parents always willing or unwilling to vaccinate all of their children? How many were they? Would it possible to analyse the reasons of such differential behavior (maybe some side-effects after vaccinating first child? Increase exposure to side-effects news? Maybe let older children decide for themselves)?

Thank you for this comment.

To understand parents with clear preferences we restricted our analysis to parents always willing or unwilling to vaccinate all of their children. This leads us to exclude 13% of the study population, 10% of which were always unsure and 3% of which held inconsistent preferences (page 3, lines 154-156).

During the survey, participants were first asked "Do you have any children of any of the following ages? (Select all that apply)" with options consisting of {Under 5, Age 5-11, Age 12-15, Age 16-17, None of the above}. For each age range selected, participants were asked the follow up question "Would you be willing to get your child(ren) of the following ages vaccinated against COVID-19?" corresponding to positive answers for any of the following {Under 5, Age 5-11, Age 12-15, Age 16-17}. As a result, the given survey questions do not allow us to investigate any sort of temporal behavior.

5) given that authors restricted the analysis the following sentence should be "Y is a binary variable for the willingness of parent I to vaccinate" ALL "their child(ren)"? Regarding Y, how the "not sure" answer were treated?

Thank you for your comment. Parents who were not sure were excluded from the analysis. We have updated the text (page 3, line 121).

6) X is equal to 1 if parent is vaccinated, please define here what vaccinated is, eg. At least one dose, two, booster.. also, define all terms that are written in the equation (eg, alpha, beta, gamma..)

Thank you for your comment. We have updated the text (page 3, lines 121-126).

7) Could it possible that only partially vaccinated parents, despite being classified as vaccinated have developed opposing view with respect to vaccination and therefore be less willing to vaccinate their children? Would it be possible to explore such hypothesis?

Thank you for your comment. We see from the results of our multivariate analysis (Figure 1 and Table 2) that being partially vaccinated is associated with willingness to vaccinate children relative to unvaccinated parents (11.9 (10.6 to 13.3)). Similarly, of the 2,697 surveyed partially vaccinated parents, just 818 (30%) are unwilling to vaccinate their children compared to 85% of the 7,654 surveyed unvaccinated parents. These results suggest that partially vaccinated parents have not developed an opposing view. We now list rates of willingness by parental vaccination status in the text (page 4, lines 167-171) and as part of a new appendix table (Appendix Table 4).

8) Equation 1, why authors did not consider any interaction effects between the vaccination status and other parent level factors? In the present form and if I understand correctly, the equation assumes that the associated effect (beta) of being vaccinated to the willingness to vaccinate is constant across any other parental difference (eg, authors are not testing if the increase in willingness to vaccinate associate with vaccinated parents is different between high income and low income parent). It seems to me that this is a strong modelling assumption that should discussed and tested.

Thank you for your comment. Results from a stratified analysis suggest that the effect of parental vaccination status on child vaccination willingness is consistent across parent level factor subgroups. We have added this analysis to the methods (page 3, lines 130-131), the results (page 4, lines 178-180), and to the appendix (Appendix Tables 6-9).

Thank you for the comment. VIF analysis suggests multicollinearity has limited effect on our regressions. In all four multivariate regressions, squared scaled GVIFs are <1.5. We now specify this in the text (page 4, lines 177-178).

10) R version, please include citation, code availability statement and package used if any

Thank you for the comment. We have included a code availability statement (page 7, line 315) and included packages used for main analysis (page 3, lines 142-144).

11) Is an ethic committee required for such type of studies? is the Boston Children's Hospital Institutional Review Board an ethics committee qualified for approval? Shouldn't be included some kind of reference/document number of the approval?

Thank you for your suggestion. We have added the IRB protocol number (page 3, lines 144-145).

Result

12) I would like to have a table reporting the actual response on willingness to vaccinate, not only the odds ratio. Contrast are given considering unvaccinated as reference, but multiple comparison may be interesting, eg is there a statistical difference between fully and partially vaccinated parents?

Thank you for the suggestions. We have added a table to the appendix (Appendix Table 4) reporting the percent willing to vaccinate their children by parental level factor. We have additionally added an appendix table (Appendix Table 5) displaying multivariate regression results, varying the reference level of parental vaccination status. We find statistical differences regardless of reference level between the subgroups and mention this in the text (Page 4, lines 175-177).

Discussion

13) If authors want to give an idea of the representativeness of their sample and generalizability of their study they may want to train their regression on a 80% subset of their data and then test the predictive performance on the other.

Thank you for this suggestion. For our regression using all parents and including parental vaccination status, we achieve an accuracy of 0.87 using a 50% cut off probability and AUC of 0.91 on a 20% held out test set. We have added this to the methods (Page 3, lines 131-133) and the results (Page 4, lines 180-182).
